# Occult recurrence of sinonasal inverted papilloma masked by a mucocele: a case report

**DOI:** 10.3389/falgy.2026.1877618

**Published:** 2026-06-26

**Authors:** Ronghua Wu, Jiangmiao Li, Yiwen Liang, Liang Tang, Yu Zou, Nan Yang, Min Li, Jian Li

**Affiliations:** 1 Allergy Center, Guangxi Hospital Division of the First Affiliated Hospital, Sun Yat-sen University, Nanning, China; 2 Department of Otorhinolaryngology-Head and Neck Surgery, Guangxi Hospital Division of the First Affiliated Hospital, Sun Yat-sen University, Nanning, China; 3 Translational Medicine Laboratory, Guangxi Hospital Division of the First Affiliated Hospital, Sun Yat-sen University, Nanning, China; 4 Guangxi Talent Highland of Promoting the Construction of National Regional Medical Center, Nanning, Guangxi, China; 5 Department of Otorhinolaryngology, The First Affiliated Hospital of Sun Yat-sen University, Guangzhou Key Laboratory of Otorhinolaryngology, Otorhinolaryngology Institute of Sun Yat-sen University, Guangzhou, China; 6 Guangxi Academy of Medical Sciences and the People's Hospital of Guangxi Zhuang Autonomous Region, Nanning, China

**Keywords:** sinonasal inverted papilloma, recurrence, mucocele, frontal sinus, diagnostic pitfall

## Abstract

**Background:**

Sinonasal inverted papilloma (SNIP) is a benign but locally aggressive tumor. Although recurrence typically presents as a solid mass on imaging, atypical manifestations may pose diagnostic difficulties.

**Case presentation:**

A 56-year-old man with previous SNIP resection presented with progressive left orbital pain. Computed tomography (CT) and magnetic resonance imaging (MRI) showed an expansile cystic lesion in the left frontal and ethmoid sinuses, initially interpreted as a mucocele. Intraoperatively, a papillomatous lesion was identified at the frontal sinus ostium. Pathology confirmed SNIP with focal mild cytologic atypia.

**Conclusion:**

SNIP recurrence can be masked by a secondary mucocele, leading to negative preoperative tests. Our experience suggests that in high-risk patients, thin-section MRI (≤1–2 mm) may improves detection, and surgical exploration with cyst wall biopsy should be performed when clinical suspicion remains high.

## Introduction

Sinonasal inverted papilloma (SNIP) is a rare benign epithelial tumor of the nasal cavity and paranasal sinuses, comprising 0.4%–7% of all sinonasal neoplasms, with an annual incidence of 0.2–1.5 per 100,000 (1). Despite its histologically benign nature, SNIP exhibits local invasiveness, a high recurrence rate (ranging from 0% to 78%) (2, 3), and a recognized risk of malignant transformation (6%–15%) (4–7). Multiple factors, including tumor location and extent, surgical technique, surgeon experience, and follow-up duration influence recurrence. Notably, involvement of the frontal sinus or the medial wall of the maxillary sinus confers a particularly higher risk of recurrence (8). Risk stratification using staging systems, such as the Krouse classification, also helps predict recurrence, with stage T3 lesions—which involve the frontal or sphenoid sinuses—carrying a notably higher risk than earlier stages (9).

Given the substantial risk of recurrence, effective postoperative surveillance is critical. Endoscopic examination is the primary modality for follow-up of SNIP, while computed tomography (CT) and magnetic resonance imaging (MRI) serve as complementary tools (10–13). On CT, SNIP typically appears as an irregular soft-tissue mass, whereas on MRI, the convoluted cerebriform pattern (CCP) is a characteristic feature (14).

Despite the availability of these imaging modalities, diagnosing recurrence can be challenging when it is masked by a secondary lesion. Here, we report a patient with SNIP in whom the recurrence was masked by a frontal sinus mucocele, leading to a diagnostic dilemma. Preoperative imaging (CT and MRI) and endoscopic examination revealed only a benign-appearing mucocele; no definitely identifiable solid component or typical cerebriform pattern was identified. Intraoperative histopathological examination of the cyst wall ultimately confirmed the diagnosis of SNIP. This case illustrates that SNIP recurrence can present as an occult process masked by a mucocele. That postoperative expansile cystic lesions in patients with a history of SNIP—despite negative findings on multiple preoperative assessments—should raise suspicion for occult tumor recurrence.

## Case presentation

A 56-year-old man was admitted with a 3-month history of progressive left orbital dull pain (Table 1). In 2020, a CT scan performed for left nasal obstruction revealed a soft-tissue lesion involving the left maxillary, ethmoid, and frontal sinuses, which was diagnosed as chronic sinusitis at that time; no surgical intervention was undertaken. In 2023, progressive nasal obstruction prompted a repeat CT scan that revealed a persistent lesion, leading to endoscopic sinus surgery at an outside institution. The surgical record indicated that functional endoscopic sinus surgery (FESS) was performed on the left maxillary, ethmoid, and frontal sinuses with removal of the lesion; however, it lacked details on attachment site management and did not confirm complete resection. Postoperative pathology confirmed SNIP involving the left frontal sinus (Figure 1), classified as Krouse stage T3. According to the patient, no regular postoperative endoscopic surveillance was performed after the 2023 surgery.

**Table 1:** Timeline of the patient's clinical course.

Time point	Event	Key findings/intervention
2020	Initial imaging	Symptoms: Left nasal obstruction 2020 CT: Soft-tissue density lesion involving the left maxillary, anterior ethmoid, and frontal sinuses Management: No surgical intervention at that time
2023	First surgery	Symptoms: Progressive nasal obstruction 2023 CT: Persistent lesion in the same locations (left maxillary, ethmoid, frontal sinuses) Management: Endoscopic sinus surgery (left ethmoid, frontal, maxillary sinuses); postoperative pathology confirmed SNIP, Krouse stage T3
2023 – early 2025	Postoperative period	According to the patient, no regular postoperative endoscopic surveillance was performed after the 2023 surgery.
2025 (preoperative)	Diagnostic workup	Symptoms: Progressive left orbital dull pain Imaging (CT and MRI): Expansile cystic lesions in left frontal and ethmoid sinuses with thinning of medial orbital wall and compression of medial rectus muscle – initially interpreted as mucoceles Biopsy: Left middle meatal biopsy showed inflammatory/polypoid changes
April 15, 2025	Second surgery	Intraoperative findings: A broad-based, friable, papillomatous lesion that bled easily was identified at the left frontal sinus ostium and recess. Frozen section: SNIP. Radical resection: Attachment-oriented radical resection was performed [complete excision of the attachment site, drilling and cauterization of the underlying bone, and wide marsupialization of the frontal sinus (Draf IIb)]. Final pathology: SNIP with mild cytologic atypia.
1 year post-surgery	Follow-up	Symptoms: Complete resolution of orbital pain. Nasal endoscopy: Patent frontal sinus ostium, smooth healthy mucosa, no evidence of recurrence.

**Figure 1:**
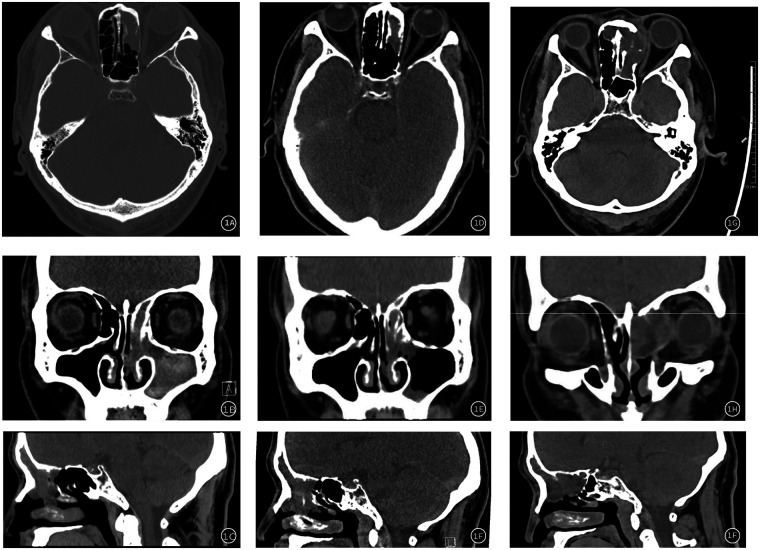
Serial CT images (axial, coronal, sagittal) of the lesion at different time points. **(A–C)** 2020: Unilateral soft-tissue density lesion involving the left maxillary, anterior ethmoid, and frontal sinuses. **(D–F)** 2023: Persistent soft-tissue density lesion in the same locations. **(G–I)** 2025: Well-defined, low-attenuation expansile cystic lesion (mucocele) in the left frontal and ethmoid sinuses, accompanied by thinning and focal bony defect of the left medial orbital wall. *Panels **(A–F)** were obtained from an outside hospital; the original scale bar was not available; image is for illustrative purposes.

In early 2025, he consulted the Department of Neurology; MRI showed cystic lesions in the left frontal and ethmoid sinuses with compression of the left medial orbital wall and medial rectus muscle. He was subsequently transferred to our department, where admission otorhinolaryngologic examination was unremarkable except for a smooth-surfaced mass with purulent drainage in the left middle meatus. Ophthalmologic examination showed best-corrected visual acuity of 0.8 bilaterally, with full extraocular movements, no proptosis, and no signs of orbital apex syndrome.

Contrast-enhanced MRI of the paranasal sinuses was performed on a 3.0-T scanner (slice thickness 4 mm). The protocol included axial T1-weighted, contrast-enhanced T1-weighted (axial, coronal, sagittal), and fat-suppressed T2-weighted (axial, coronal) sequences. The images showed a well-defined expansile cystic lesion in the left frontal and ethmoid sinuses, with thinning and focal defect of the left medial orbital wall and compression of the medial rectus muscle. On T1-weighted images the lesion was isointense; on contrast-enhanced T1-weighted images it showed peripheral rim enhancement without a definitely identifiable solid component; and on fat-suppressed T2-weighted images it was hyperintense. These findings were initially interpreted as a benign mucocele (Figure 3). A preoperative biopsy of the left middle meatal lesion showed no evidence of SNIP (Figure 2). These findings were initially interpreted as mucoceles. Given the patient's history of high-risk SNIP (Krouse stage T3) and persistent orbital symptoms, endoscopic sinus surgery was performed.

**Figure 2:**
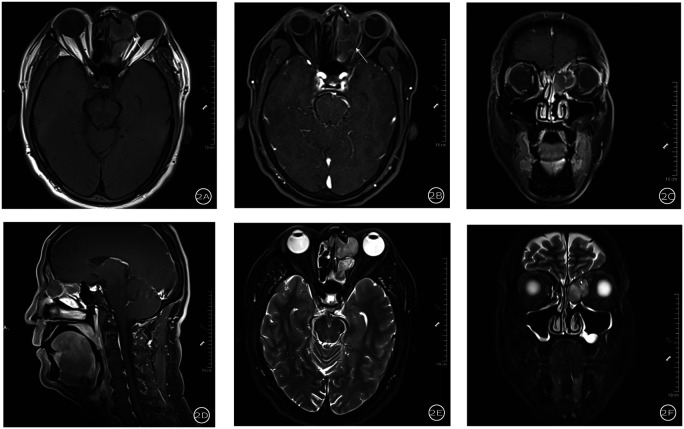
Preoperative MRI of the left frontal and ethmoid sinuses. **(A)** Axial T1-weighted image shows a well-defined, isointense lesion in the left frontal and ethmoid sinuses, with bony defect of the left medial orbital wall and mass effect on the medial rectus muscle. **(B)** Axial contrast-enhanced T1-weighted image demonstrates low-to-intermediate signal intensity with peripheral rim enhancement, well-defined margins, persistent medial orbital wall defect, and medial rectus compression. **(C)** Coronal contrast-enhanced T1-weighted image shows the lesion protruding into the left orbit, with peripheral rim enhancement and no invasion of the globe. **(D)** Sagittal contrast-enhanced T1-weighted image confirms similar features: well-defined borders, low-to-intermediate signal, and rim enhancement. **(E)** Axial fat-suppressed T2-weighted image reveals high signal intensity of the lesion, well-defined margins, medial orbital wall defect, and medial rectus compression. **(F)** Coronal fat-suppressed T2-weighted image shows the high-signal lesion extending into the orbit without globe invasion.

**Figure 3:**
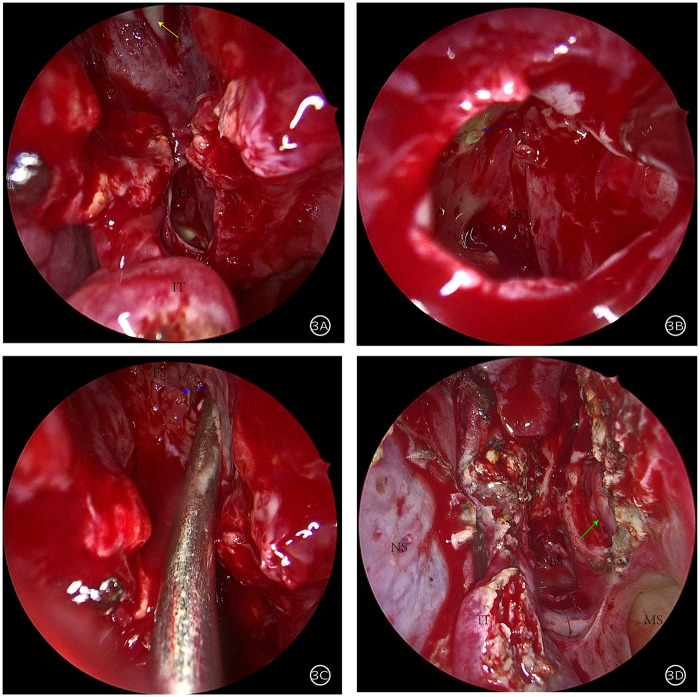
Intraoperative findings. **(A)** Thick, white, mucopurulent secretions within the left frontal sinus (yellow arrow). **(B)** Expansile cystic lesion in the left posterior ethmoid sinus containing yellowish, thick mucoid material (black arrow). **(C)** At the left frontal recess and frontal sinus ostium, irregular, granular, papillomatous mucosa with a broad base of attachment (blue arrow). **(D)** A bony defect of the left medial orbital wall with intact underlying periorbital fascia was observed (green arrow). *Approximate scale is indicated by the endoscopic instrument diameter (2 mm).

On April 15, 2025, under general anesthesia with intraoperative navigation, a left frontal sinus mucocele with purulent drainage and partial lamina papyracea defect was identified. At the left frontal recess and frontal sinus ostium, a broad-based, friable, papillomatous lesion that bled easily was observed (Figure 4). Intraoperative frozen section confirmed the diagnosis of SNIP with focal mild cytologic atypia (Figure 2). Therefore, attachment-oriented radical resection was performed: the precise mucosal attachment site at the frontal recess was excised, the underlying bone drilled and cauterized, and the frontal sinus widely marsupialized (Draf IIb). Paraffin section pathology confirmed SNIP as well (Figure 2).

**Figure 4:**
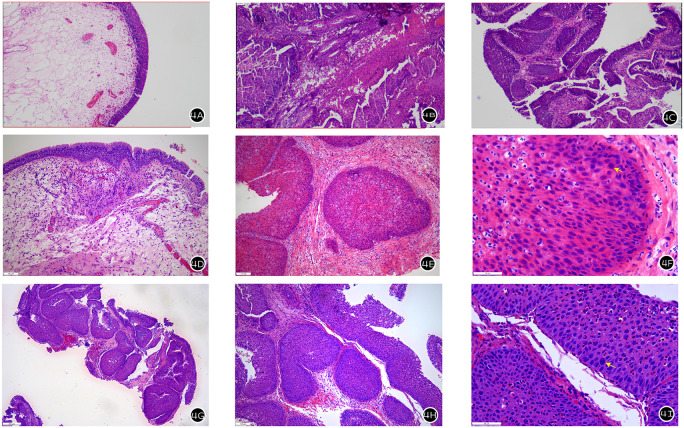
Histopathological findings at different time points (H&E stain). **(A–C)** 2023 postoperative specimens (left sinonasal cavity, exact subsite not specified): **(A)** Chronic inflammatory changes; **(B,C)** Sinonasal inverted papilloma (SNIP). **(D)** preoperative biopsy (left middle meatus): Inflammatory and polypoid mucosal changes with no definite evidence of SNIP. Original magnification:   ×  100. **(E,F)** 2025 intraoperative frozen sections (left frontal recess/ostium): features consistent with SNIP. Mild cytologic atypia is indicated in panel F (yellow arrow). Original magnification: **(E)**  ×  100; **(F)**  ×  400. **(G–I)** 2025 permanent sections (same lesion) showed inverted sinonasal papilloma with squamous epithelial hyperplasia, inflammatory cell infiltration, and mild cytologic atypia (yellow arrow in I). Original magnification: **(G)** × 50; **(H)** × 100;**(I)** × 400. *(A–C) Original magnification not available (external hospital slides).

The patient's orbital pain resolved completely within 2 weeks after surgery. At the 1-year postoperative follow-up visit, nasal endoscopy showed a patent left frontal sinus ostium with smooth, healthy mucosa and no evidence of tumor recurrence.

## Discussion

In this case, comprehensive preoperative evaluation—including CT and MRI, endoscopic examination, and middle meatal biopsy—failed to detect the underlying SNIP, showing only a benign-appearing mucocele. Despite the absence of any positive finding, the patient ultimately had histopathologically proven SNIP with mild cytologic atypia.

### Why did preoperative assessments fail?

Several factors explain this. First, the papillomatous tissue was focal and confined to a small area on the cyst wall, rather than forming a solid mass, likely due to compression by the secondary mucocele. Second, based on the principle of partial volume averaging, a standard 4-mm MRI may miss a focal lesion confined to the cyst wall; thin-slice (≤2 mm) MRI has been shown to improve diagnostic accuracy (15–17). Therefore, thin-section MRI should be considered for SNIP patients when occult disease is suspected. The absence of thin-section imaging is a limitation of our preoperative workup. Third, postoperative changes—including mucosal thickening, scarring, and inflammation—can obscure typical SNIP features, such as the CCP, leading to false negatives in the postoperative setting (14, 18). Finaly, the preoperative biopsy was taken from the middle meatus, not from the frontal recess/ostium where the true lesion resided. Angled endoscopy was used, but deep sampling was not tolerated under local anesthesia. Image-guided biopsy was not performed because preoperative imaging showed no definable target, and it is not standard for office-based procedures. These factors explain the false-negative result.

### Residual disease or true recurrence?

Residual SNIP is defined as an incompletely resected tumor that persists after surgery, whereas true recurrence denotes new tumor growth after complete resection (19). In our case, whether the initial surgery achieved complete resection is unknown, and the lesion reappeared within two years at the same site; thus, residual disease cannot be excluded. However, the 2023 specimen showed no atypia, whereas the 2025 specimen demonstrated focal mild cytologic atypia – a finding that suggests true biological progression. In clinical practice, distinguishing between residual disease and true recurrence is often difficult, and the term “recurrence” in the literature has long encompassed lesions that may have originated from residual disease (20, 21). Given this convention and the evidence of pathological progression in our case, we refer to the lesion as “recurrent” while acknowledging the possibility of residual origin. Importantly, this distinction does not alter the clinical message: high-risk SNIP patients warrant long-term surveillance regardless of classification.

#### Surveillance recommendations

Endoscopic examination is the cornerstone of follow-up (22); CT/MRI are complementary for high-risk features (18, 22–24). Most recurrences occur within 2–3 years (89% within 2 years) (20, 25), but late recurrences mandate long-term follow-up (26). A minimum of five years of surveillance is recommended (22, 27). Patients with high-risk features—frontal sinus involvement, high-grade dysplasia, or advanced T-stage—may benefit from lifelong follow-up (8, 28). Dysplasia significantly increases recurrence risk (38.1% vs. 17.2% in a multi-institutional study (29). In our case, SNIP with focal mild cytologic atypia (not present originally) signals progression and justifies intensified surveillance.

## Strengths and limitations

While a few cases of SNIP associated with a mucocele have been reported, all share a common feature: the coexistence of the tumor and the mucocele was evident on preoperative imaging, and the focus of these reports was either the unusual clinical presentation or the surgical technique used (30, 31). To our knowledge, this is the first report that highlights the diagnostic pitfall of a completely negative preoperative workup in a patient where a secondary mucocele entirely masked an occult recurrence of SNIP with pathological progression. This case therefore provides a unique educational message for clinicians managing high risk SNIP patients. Nevertheless, several limitations should be acknowledged. Clinical follow-up is currently restricted to one year (simply because only one year has elapsed since surgery); longer-term surveillance is needed. In addition, some data were obtained from an outside hospital, limiting our ability to provide scale bars or original magnifications.

## Conclusion

In summary, this case underscores that SNIP may be masked by a postoperative mucocele, that imaging and biopsy may be falsely negative in challenging anatomical sites (suggesting the use of thin-section MRI for better detection), and that long-term surveillance is critical for high-risk patients. An endoscopic exploration with histopathology is recommended when clinical suspicion remains high.

## Patient perspective

The patient reported that his preoperative left orbital dull pain significantly affected his daily activities. After surgery, his symptoms resolved completely. He understands the importance of regular endoscopic follow-up and has agreed to long-term surveillance.

## Data Availability

The raw data supporting the conclusions of this article will be made available by the authors, without undue reservation.
